# Blueprint 2: A self-directed mobile adaptive coping skills intervention to improve psychological distress symptoms among cardiorespiratory failure survivors study protocol

**DOI:** 10.1016/j.cct.2026.108359

**Published:** 2026-05-30

**Authors:** Robin L. Baudier, Katie E. Mosack, Jodi A. Lapidus, Laura S. Porter, Aliza Anderson, Aluko A. Hope, Allison Johnson, Kristy Johnson, Kathryn Lesowski, Rafaela Avallone Mantelli, Carolyn J. Marentes Ruiz, Madeline McDougal, Luz Mejia-Flores, Julie Mitchell, Marc Moss, Thanh H. Neville, Cirila Estela Vasquez Guzman, Annette Vu, Edlyn L. Wolwowicz, Catherine L. Hough, Cynthia D. Morris, Christopher E. Cox

**Affiliations:** a Biostatistics and Design Program, Oregon Health & Science University, Portland, OR, USA; b Oregon Health & Science University-Portland State University School of Public Health, Portland, OR, USA; c Oregon Clinical and Translational Research Institute, Oregon Health & Science University, Portland, OR, USA; d Department of Psychiatry & Behavioral Sciences, Duke University, Durham, NC, USA; e Department of Medicine, Division of Pulmonary, Allergy, & Critical Care Medicine, University of Colorado, Denver, CO, USA; f Department of Medicine, Division of Pulmonary and Critical Care Medicine, Oregon Health & Science University, Portland, OR, USA; g Department of Medicine, Division of Pulmonary & Critical Care Medicine and the Program to Support People and Enhance Recovery (ProSPER), Duke University, Durham, NC, USA; h Department of Medicine, Division of Pulmonary Critical Care, University of California, Los Angeles, Los Angeles, CA, USA; i Department of Family Medicine, Oregon Health & Science University, Portland, OR, USA; j Department of Medicine, Oregon Health & Science University, Portland, OR, USA

**Keywords:** Critical illness, Intensive care units, Psychological distress, Depression, Anxiety, Adaptive coping skills training

## Abstract

**Introduction:**

Persistent psychological distress including symptoms of depression, anxiety, and posttraumatic stress disorder (PTSD) occur in as many as 70% of intensive care unit (ICU) survivors. However, few therapies exist that target this population's unique symptoms and needs. To address this, we developed the Blueprint adaptive coping skills mobile app, which was found to be feasible and reduce psychological distress symptoms in a recent single-center pilot randomized control trial (RCT).

**Objective:**

To describe the methods of the Blueprint 2 multi-center RCT (clinicaltrials.gov ID NCT06538246) being conducted to test the efficacy of the Blueprint app in reducing psychological distress among ICU survivors compared to an education control in a geographically and sociodemographically diverse population, including Spanish-language speakers.

**Methods and analysis:**

In this RCT, 400 participants with a baseline Hospital Anxiety and Depression Scale (HADS) total score ≥8 will be randomized in a 1:1 ratio to intervention or control. The HADS total score at one month post-randomization is the primary outcome. Secondary outcomes include HADS anxiety and depression subscales, posttraumatic stress scale (PTSS), and the quality-of-life EQ-5D 100-point visual analog scale (VAS) at 1, 3, and 6 months. Select subgroup analyses including users of the Spanish-language version of the app will be used to evaluate consistency of effects across patient populations. Constrained longitudinal data analyses will compare outcomes across intervention groups and timepoints.

## 1. Introduction

Cardiorespiratory conditions such as acute respiratory distress syndrome (ARDS), congestive heart failure, myocardial infarction, pneumonia, and sepsis are among the most common ICU diagnoses, as well as the most common causes of morbidity and mortality [1]. Those who survive critical illness also frequently develop the Post-Intensive Care Syndrome (PICS), which consists of impairments across physical, cognitive or mental health domains such as fatigue, insomnia, cognitive dysfunction, depression, anxiety, and posttraumatic stress disorder (PTSD) [2,3]. Psychological distress symptoms are particularly common, with prevalence estimates of PTSD, depression and anxiety ranging from 22% to 70%, and can persist for months to years [4–10]. These symptoms are associated with cognitive impairment [11–13], functional status limitations [14,15], frailty [16], as well as worse cardiorespiratory outcomes including mortality [17–29]. As such, psychological and emotional well-being is central to quality of life [30–33].

**Fig. 1.**
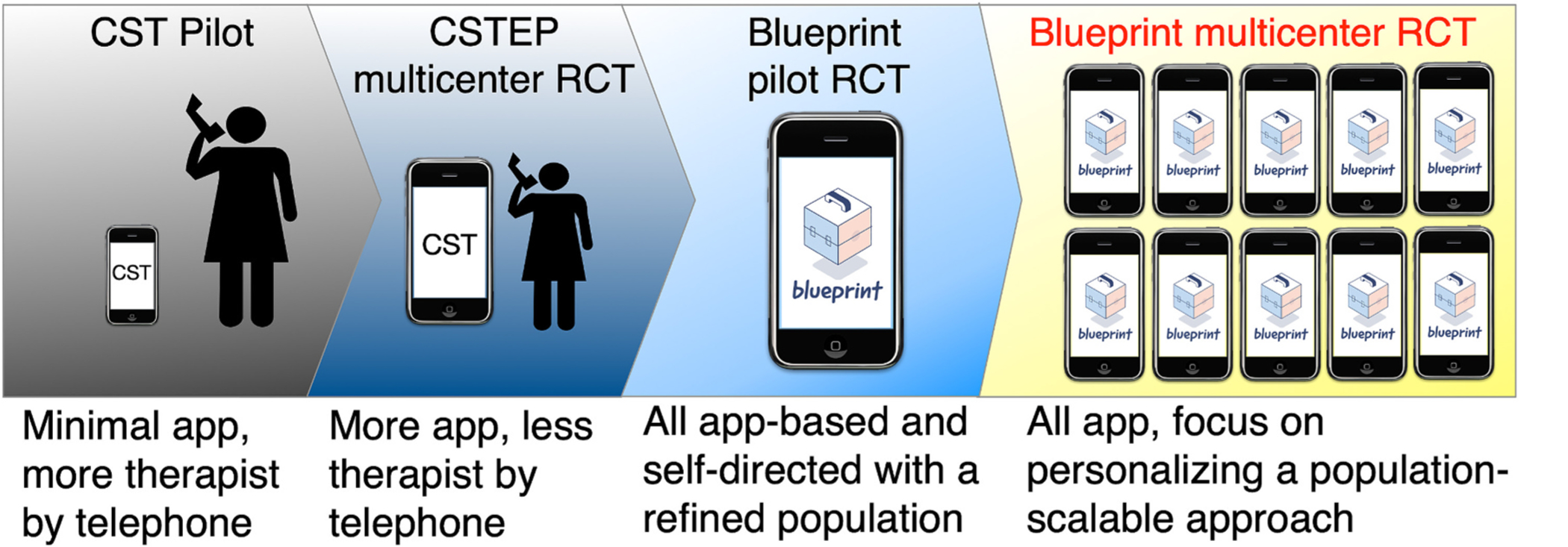
The Blueprint adaptive coping skills training (CST) intervention evolution across studies. The first Blueprint adaptive coping skills training (CST) intervention pilot study involved minimal app usage with substantial phone calls from a therapist. The following multicenter randomized control trial (RCT) with education program control (CSTEP) had less therapist involvement and more app usage. The current Blueprint intervention is an all app-based and self-directed intervention, which was first piloted at a single site and is now expanded to a multi-center RCT.

A major barrier to addressing these symptoms among ICU survivors is the lack of evidence-based interventions that are easy to access and relevant to their experience [34]. An additional challenge is the historically limited representation in RCTs from patients from certain racial and ethnic communities, including people whose first language is not English [35]. To fill this gap, we developed Blueprint, an adaptive coping skills training (CST) intervention. We have optimized Blueprint through years of research, from a months-long telephone-based program tested in a single center pilot study [36], to a telephone- and web-based version tested in a multicenter RCT (CSTEP) [37], to a completely self-directed and mobile app-based version evaluated in a recent Blueprint pilot RCT [38] (Fig. 1). Throughout these studies, the intervention has consistently reduced symptoms of distress among patients with elevated baseline symptoms. In this study, the fully app-based Blueprint intervention will undergo a formal test of efficacy for the first time in a population that is geographically diverse and includes English- and Spanish-language speakers, the latter of whom will be utilizing our first Spanish-language version of the intervention. Here, we describe the design, methods and planned efficacy analyses of the Blueprint 2 RCT (clinicaltrials.gov ID NCT06538246).

## 2. Materials and methods

### 2.1. Human subjects and regulatory oversight

All procedures for Blueprint 2 RCT, including informed consent, were approved by the Duke University central IRB (cIRB, Protocol ID Pro00115623) and approved by local IRB sites at Oregon Health & Science University (OHSU), the University of Colorado (CU), and the University of California- Los Angeles (UCLA) prior to study initiation. An independent data safety and monitoring board (DSMB) was established prior to study initiation to review and approve the protocol and safety monitoring procedures; subsequent reviews of study activities and data collection occur semi-annually. For this study, adverse events (AEs) are defined as any new HADS score ≥ 18 when previous scores were < 18 and any suicidal ideation that is reported to study personnel. Serious adverse events (SAEs), including those that are fatal; life-threatening; persistent, significantly disabling, or incapacitating; that result in inpatient hospitalization or emergency room visits exceeding 24 h in duration; and/or create a persistent or significant disability will be reported to the DSMB, the cIRB, and National Heart, Lung, and Blood Institute (NHLBI) program officer.

### 2.2. Study objectives

The aims of this study are to test the efficacy of Blueprint on improving symptoms of depression, anxiety, PTSD, and quality of life among cardiorespiratory failure survivors and to determine which patient-level characteristics are associated with a greater treatment response among sociodemographic subgroups of interest.

### 2.3. Study design

**Fig. 2.**
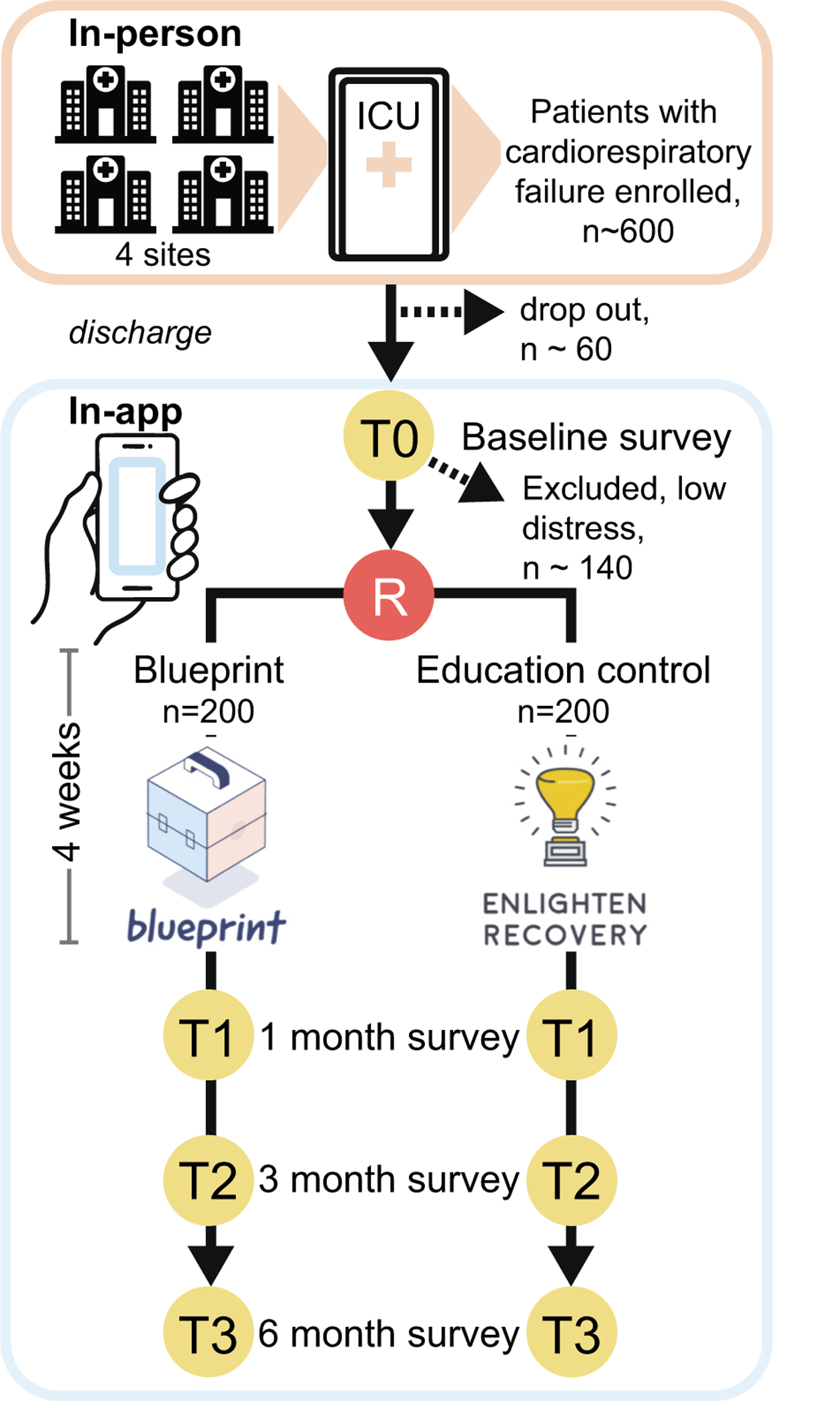
Blueprint 2 study flow diagram. Intensive care unit (ICU) patients with cardiorespiratory failure are being recruited and enrolled in person across 4 study sites with determination of final eligibility, randomization, intervention, and follow-up all completed by the participant in-app after hospital discharge. The duration of the study is 6 months from randomization (R), which includes the four-week intervention (Blueprint intervention or Enlighten Recovery control), and 1 (T1), 3 (T2), and 6 (T3) month follow-up surveys.

This five-year, multi-center study employs a parallel-group, RCT design to evaluate the efficacy of Blueprint, a four-week behavioral intervention in a geographically diverse and English- or Spanish-speaking population (Fig. 2). Participants are randomly assigned to either the intervention group (Blueprint) or to the control group (Enlighten Recovery), which receives educational materials only [39]. The duration of the study for participants is 6 months from randomization (i.e., baseline), with data used in primary and secondary analyses collected at baseline, 1 month (primary endpoint), 3 months, and 6 months post-randomization. Data will also be collected weekly during the 4-week intervention (at the end of weeks 1, 2, 3, and 4) to trigger additional content for participants with severe or increasing HADS scores and will be used in sensitivity analyses. Both intervention and control content are delivered and outcome data are collected through the same mobile app platform. From the date of first patient enrolled, we estimate it will take 41 months to complete data collection (35 months to enroll 600 participants with an additional 6 months to complete follow-up) and 8 months to perform all final data analyses (primary and secondary outcomes models followed by exploratory and sensitivity analyses) and manuscript preparation.

### 2.4. Recruitment and eligibility

Recruitment began October 15, 2024; as of manuscript publication date, recruitment is ongoing with a planned end date of October 15, 2027. Participants are being recruited from hospital ICUs affiliated with the following academic centers: Duke, OHSU, UCLA, and CU. Clinical research coordinators (CRCs) use local electronic medical records (EMRs) to screen patients in ICUs and stepdown units for potential eligibility and then approach for recruitment before hospital discharge. During the screening and consent process, the CRC provides an overview of the trial, shows a 2-min animated video created to explain the trial, and, if the patient is interested in participating, obtains their informed consent. We plan to consent 875 individuals to randomize 400 participants. Eligibility criteria, which is assessed in two stages (i.e., before and after discharge) can be found in Table 1.

### 2.5. Experimental condition allocation

Within 48 h post-discharge, all enrolled participants are prompted by the mobile app to complete the HADS survey within the app. Patients with elevated HADS total scores (≥8) are stratified by language (English or Spanish) and auto-randomized within language group by the platform in a 1:1 group ratio using an adaptive, biased-coin minimization method to balance study site and the HADS total scale score < 20 vs. ≥20 in treatment group allocation [40]. This algorithm is programmed directly within the mobile app platform; details of the algorithm are in Appendix A of the Supplemental Material. After completing the baseline survey, the assigned Blueprint or Education Program content auto-populates the app platform and prompts program initiation.

### 2.6. Participant engagement and retention plan

The mobile app platform used by both groups has built-in system-generated mechanisms to prompt participants to complete app-related activities. Within 48 h of discharge, participants receive a notification on their device to start using the application. The self-directed program delivers timed alerts on the device home screen to prompt use thereafter. Following the four-week intervention, participants in both arms receive messages to complete 1, 3, and 6 month surveys and every week thereafter until the survey is completed. Although participants can choose to remove the app from their mobile phones, unless they have requested to withdraw or temporarily change to an inactive status, they continue to receive automated text notifications. For participants who fail to initiate or continue app usage, CRCs are prompted to follow up using a protocolized text- and phone-based strategy.

### 2.7. Blueprint intervention

The Blueprint intervention is divided into four week-long blocks focusing on different cognitive-behavioral strategies to assist participants in coping with post-ICU challenges, including feelings of anxiety, depression, and isolation. The content is arranged thematically across the four weeks, a duration shown to be equally efficacious as longer programs [41,42]. Participants are guided to create personalized action plans to aid in their gradual reintegration into daily activities, emphasizing both physical rehabilitation and psychological well-being.

Weekly themes: Week 1 includes *progressive muscle relaxation* as a way to cope with stress, pain, and fatigue and *pleasant imagery* to aid in managing emotional distress related to physical symptoms [43]. During Week 2, participants are encouraged to gradually increase activity through *activity-rest cycling* in which demanding activities are broken up into periods of moderate activity and rest [44], and to engage in *pleasant activity planning*. During Week 3, participants learn how to *cope with unhelpful automatic thoughts* during recovery by recognizing the relationship between thoughts, feelings, and behavior and then replacing unhelpful thoughts with more positive thoughts. A brief in-the-moment relaxation technique called *fast relax* is also taught. During Week 4, patients are trained in a systematic method of problem-solving and developing plans for *using Blueprint skills in real life situations* [45].

**Fig. 3.**
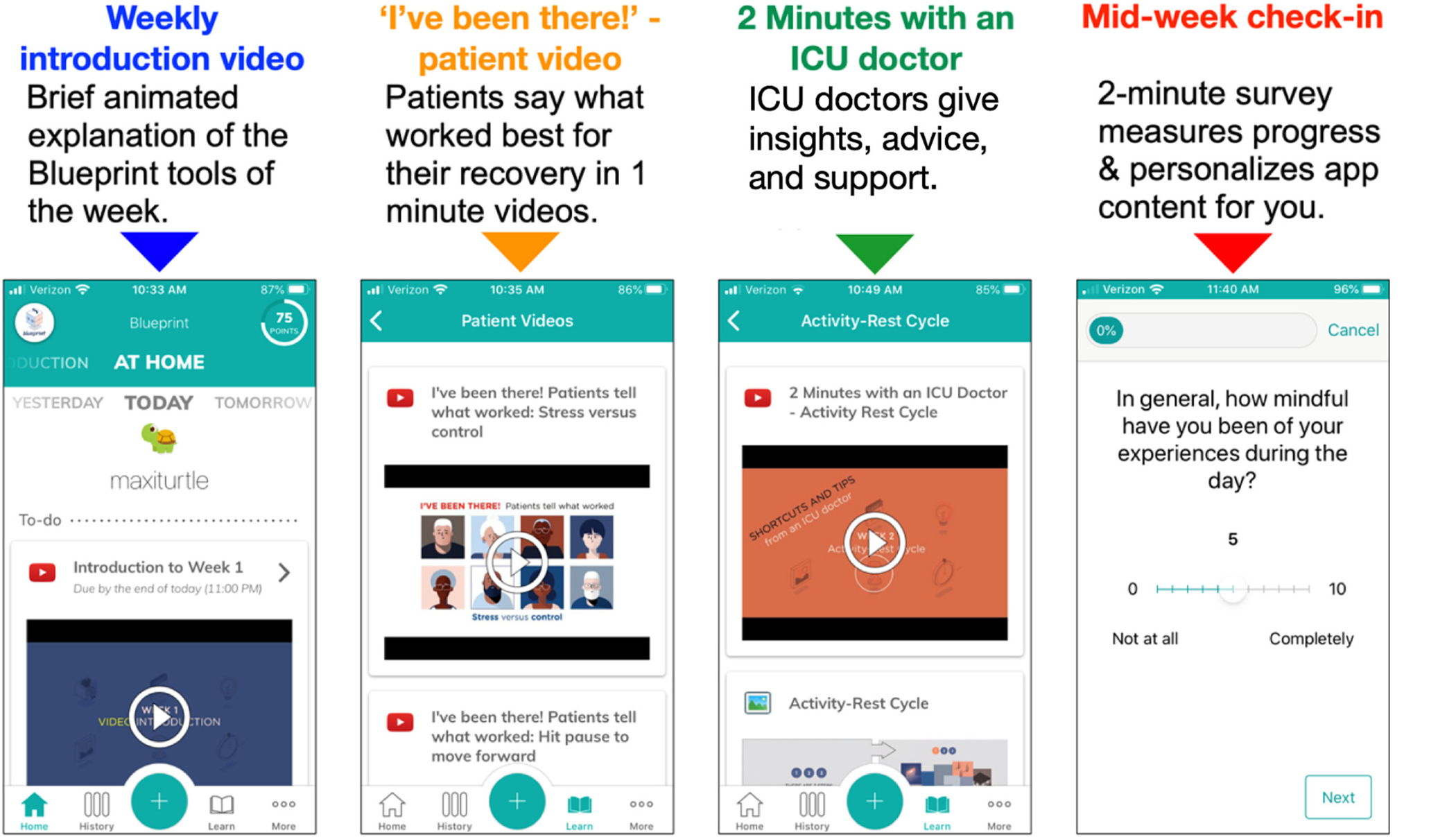
Screenshots of core types of Blueprint platform features. Core features include weekly introduction videos, patient videos, two minutes with an ICU doctor, and a mid-week check-in.

**Fig. 4.**
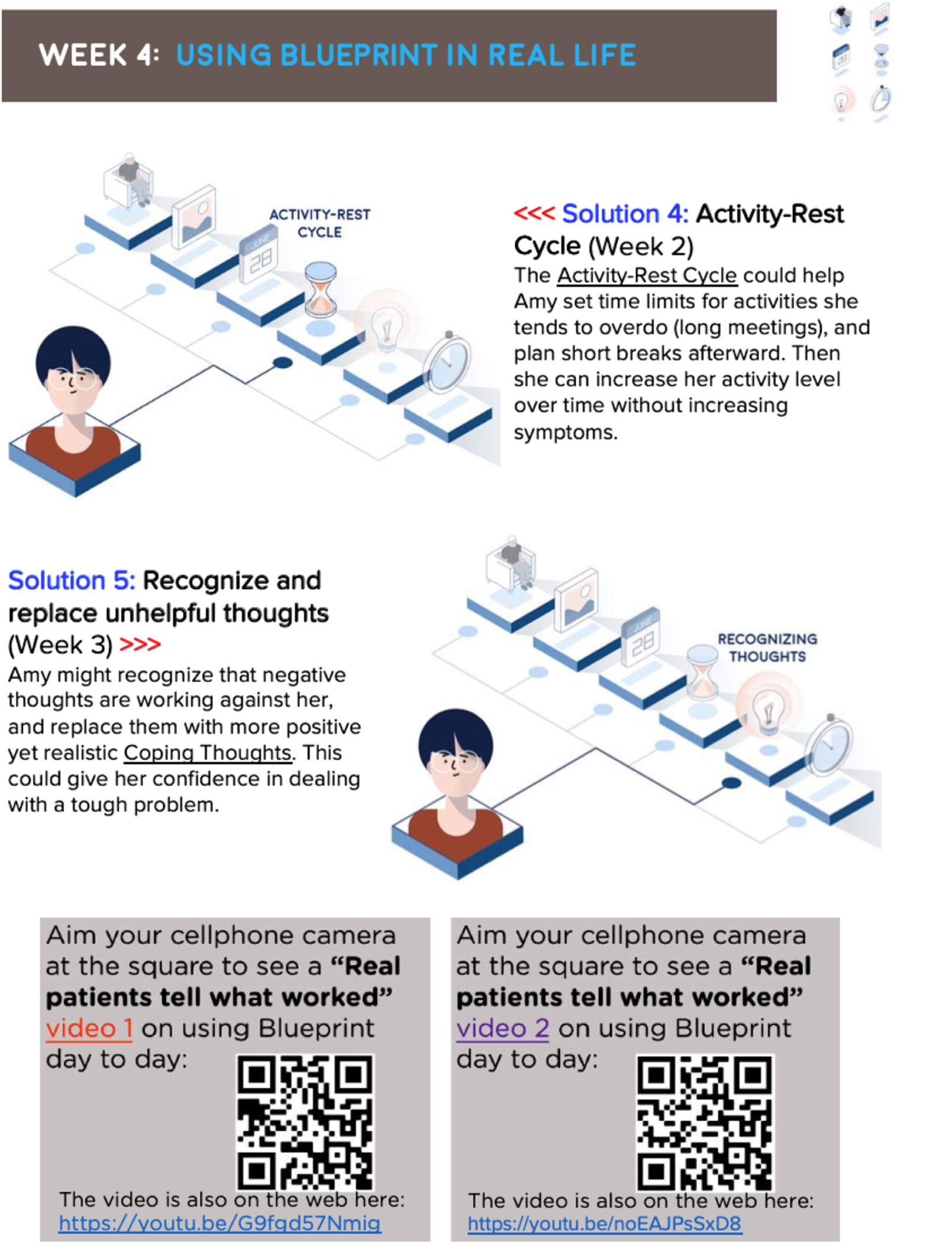
Example page from the Blueprint workbook. A 36-page Blueprint workbook provides complementary material, exercises, and QR code links to app content.

Content: Each week's key concepts are introduced by an animated video (Fig. 3). Actual patients who have previously been hospitalized for cardiorespiratory failure describe coping strategies (the “I've Been There” feature) in 2–4 brief videos each block, each approximately one minute in length. In the “2 Minutes with an ICU Doctor” segment, clinicians contextualize adaptive coping within recovery and normalize the process. Each week's coping skills are illustrated through interactive exercises that mimic common daily situations and stressors. The ‘mid-week check-in’ prompts a HADS survey, with ~1 min video content recapping the week's content and encouraging participants to practice it displayed in response to severe (>15) or increasing (i.e., higher than the prior week) scores. Overall, participants are prompted to engage in primary intervention activities 2 to 4 days each week with no single activity requiring more than 10 min. In addition to the primary app content, supplementary app activities are made available, including additional video content and guided practices. A 36-page Blueprint workbook sent to participants post-randomization provides complementary material, exercises, and QR code links to app content (Fig. 4), mailed to each recipient and available as a PDF.

### 2.8. Education control

The control arm, which we call Enlighten Recovery, is an education program [37,46] we developed that contains cardiorespiratory condition-specific content, informed by research on informational needs and past successful education programs [47–49]. Participants who have been randomized to this arm are prompted via automated alerts to use the mobile app. Participants receive educational materials about cardiorespiratory conditions, recovery, and health management post-ICU; this information does not incorporate specific behavioral strategies or support mechanisms. Each week, participants are directed to watch a 3 to 5 min informational video related to the week's content and to complete the HADS survey. The time commitment for the Enlighten Recovery arm is less than the Blueprint intervention by approximately 20 min per week. Similar to the Blueprint intervention, a printed or PDF workbook with QR links to additional videos and educational materials related to each of the four weekly topics is also mailed to participants post-randomization.

### 2.9. Spanish-language adaptation

All materials and activities (including concept and behavioral skills videos, videos featuring real patients and ICU physicians related to post-ICU coping for those in the intervention arm and educational materials in the control arm) were adapted into a Spanish-language version with the goal of being easily understandable and culturally relevant for Latin American Spanish speaking audiences. We also translated the workbooks, intervention website, and all patient-communication media (e.g., participant guides for the app, welcome postcards), which are delivered by Spanish bilingual CRCs or CRCs in conjunction with interpreters. The translation of content was performed in an iterative three-step process by a multidisciplinary team of native English- and Spanish-language speakers. The process involved an initial cultural and linguistic adaptation of key scripted content by an external group of community members and clinicians, creation of final versions of material by bilingual trial network members, and formal usability testing among bilingual participants. This adaptation process will be further enumerated in a future paper.

### 2.10. Outcome measures

Outcome data collection for randomized participants occurs at four timepoints as shown in Table 2: at randomization (i.e., baseline) as well as 1, 3, and 6 months post-randomization. The HADS total score at 1 month is the primary outcome of the trial. The HADS is a self-assessment questionnaire (range 0–42) assessing both anxiety (HADS Anxiety subscale) and depression (HADS Depression subscale) [50]. Additionally, the HADS is administered to both groups in the second half of each week of the 4-week intervention through the app during days 4 to 6, 11 to 13, 18 to 20 and 25 to 27. Scores from participants who complete the HADS after completing Week 4 are used as the Month 1 measure. Any participant who misses the Week 4 HADS measure are prompted to complete a month 1 HADS on day 30. Collection windows for Month 1 and Month 3 timepoints end the day before collection begins for the next timepoint and end two months after collection begins for the (final) 6-month timepoint.

Secondary outcomes include differences in the HADS total score at months 3 and 6, as well as the following measures completed at 1, 3, and 6 months: the HADS Anxiety and Depression subscales [50]; PTSS, used to assess ICU-related trauma (i.e., PTSD) by anchoring memory recall to hospitalization [51]; the Modified Patient Health Questionnaire-15 physical symptom scale (mPHQ-15), which is a ten-item version of the PHQ-15 measure of physical symptoms [52] previously adapted for post-ICU patients [53]; and the EQ-5D 100-point visual analog quality of life scale. See Supplementary Material Appendix B for detailed descriptions of outcomes.

### 2.11. Data collection and management

Clinical characteristics collected through medical chart review are entered by CRCs in electronic case report forms securely integrated within the REDCap data management system [54,55] and include: insurance status, highest level of care environment (ICU or stepdown unit) during hospitalization, component questions to derive Charleston Comorbidity Index [56], ICU primary admission diagnosis, highest level of respiratory failure support present during hospitalization, surgery during hospitalization, psychiatric and pain medications, illness severity (Acute Physiology And Chronic Health Evaluation II [APACHE II] component questions and score) [57], and length of hospital stay. All other data collected are entered by participants directly in the Blueprint app. In addition to outcome data, these measures include: date of birth (used to calculate age), address (used to derive area deprivation index [58]), marital status, sex, race, ethnicity, primary language, presence of instrumental support, presence of minor children in the home, work status the month before hospitalization, and highest grade completed in school. See Supplementary Material Appendix B for detailed descriptions of all study assessments.

The data coordinating center (DCC) at OHSU uses REDCap to merge study data collected directly with biweekly data transmitted securely from the app platform. Only DCC data managers can view all patient-level data and experimental conditions. Intervention arms are masked for statisticians until final analysis. DCC data managers actively monitor all data throughout the study, identifying records needing attention or correction (e.g., missing, outlier, discrepancy) by CRCs. The DCC sends a bimonthly recruitment and retention progress to study staff, which is discussed at regularly scheduled meetings.

### 2.12. Statistical analysis

Outcomes as well as patient characteristics will be characterized by group at baseline by mean (SD), median (IQR), and N (%), as appropriate. Analyses of primary and secondary outcomes will be performed utilizing constrained longitudinal data models (cLDA), which are mixed effects models where both groups are constrained to have the same baseline intercept. As recommended to improve precision [59,60], the model will be adjusted for randomization variables of site and primary language spoken. Random effects for subject and an unstructured covariance structure will account for correlation between repeated measures. Model estimates will include a shared baseline intercept, estimates for difference from baseline for 1–6 months, and estimates for difference between Blueprint and Education Control at 1–6 month timepoints. Estimates will be reported with a 95% CI and *p*-values will be used to determine significant differences.

In secondary subgroup analyses utilizing cLDA models as described above on subsets of participants, we will examine whether the intervention effects are consistent or vary in magnitude across pre-specified sociodemographic subgroups that might have comparatively less access to therapy on average: Black race, Hispanic ethnicity, and participants with Spanish as a primary language (i.e., participants using the Spanish-language version of the app). Sensitivity analyses will be performed to estimate how much loss to follow-up may have affected primary outcome effect estimates using cLDA models with 1-month values imputed from linear regressions of any available weekly HADS scores collected during the intervention.

### 2.13. Sample size and power considerations

We estimated power based on means, standard deviations, and within-patient scale correlations over 6 months derived from our recent RCTs conducted with >500 patients as well as from the recent literature [37,38,46,53,61]. We originally planned to enroll 530 participants to randomize 400. However, due to a lower observed than estimated randomization rate in enrollment after study initiation (45.7%), we increased the target enrollment. We now estimate we will need to enroll ~875 patients to sufficiently safeguard against dropout before baseline and low post-discharge HADS scores at baseline to randomize 400. We conservatively assume 19% cumulative post-randomization dropout by 1 month, leaving 324, as well as an additional 15% dropout between 3 and 6 months [37,38,46]. Assuming a baseline standard deviation of 8.7 and correlation between baseline and 1 month of 0.4, this sample size will provide 90% power with an alpha of 0.05 to detect a treatment group difference of 2.6 units in the 3 month HADS total score, which represents an enduring effect difference similar to the minimal clinically important difference [62–64] in a comparison of two means via repeated-measures analysis. This will also provide 99% power with an alpha of 0.05 to detect a 2-unit difference in the primary outcome, the HADS total score at 1 month, as well as a 3.8-unit difference in the PTSS secondary outcome [61,65]. For subgroup analyses, in a comparison of two means in a repeated-measures analysis between baseline and 3 months, assuming a standard deviation of 4.5 and correlation between baseline and 3 months of 0.5, we will have 80% power with an alpha of 0.02 (to reasonably account for multiple comparisons) to detect group differences in the HADS total score of 4.2 units with a subgroup n of 100, 5.6 units with an n of 80, and 6.0 units with an n of 60, effects similar to those seen in the Blueprint 1 pilot RCT [38]. Power calculations were conducted via the comparison of two means in a repeated measures design in PASS (NCSS, Kaysville, UT).

## 3. Discussion

The Blueprint 2 RCT evaluates the impact of a mobile health, adaptive coping skills training intervention on psychological well-being and quality of life following ICU discharge among patients with serious cardiorespiratory conditions. While other investigators have developed interventions designed to improve post-ICU psychological outcomes [66–68], this remains an overlooked area of research. This study is further distinguished by its use of mobile health technology to deliver comprehensive intervention content to ethnically and linguistically diverse patient populations. The intervention incorporates several precision-oriented approaches, including the introduction of weekly themes which are augmented by multimodal points of contact provided by the app interface, along with additional resources for those who indicate increasing or severe distress. The study uses a multi-functional mobile app platform that contains the randomization algorithm, delivers intervention content, and supports self-reported data collection for all outcomes. Particularly novel is the ability to identify and target high-risk patients through the app for augmented intervention dosing – an adaptive approach that could be utilized in future studies for other targeted interventions. App analytics provide detailed data on participant engagement with content, which enable CRCs to follow up with the participants in real time to reduce participant attrition and will enable data analysts to explore the relationship between participant engagement and intervention effects.

One potential limitation of our intervention is that the app platform might be challenging for participants who struggle with mobile phone navigation. We have attempted to address these issues by assisting patients with app set-up before discharge and by sending them home with a postcard containing contact numbers for support. A known limitation of our study is that it does not estimate the intervention's effects in the sickest of ICU patients due to the challenges in retaining participants with ongoing severe health challenges, which may limit the generalizability of these findings to more severely ill ICU patients. To improve generalizability of findings, we have adapted the intervention to Spanish-language speakers and have selected sites to maximize geographic and demographic diversity. However, though we can assess generalizability through subgroup analyses, app usage and intervention effectiveness may differ in a real world setting including broader populations and without CRC set-up assistance and follow-up. We have used a three-step iterative approach to translate the Blueprint intervention to Spanish with the goal of making the translation easily understandable and culturally relevant to a Latin American Spanish-speaking population, but it is possible we may not have adequately captured culturally relevant clinical and psychological concepts given the heterogeneity of Hispanic and/or Latinx populations.

Our work to date has been focused on identifying a patient-friendly and cost-efficient intervention that addresses the psychological sequalae of having survived an ICU admission for cardiorespiratory distress. With this large-scale multi-site RCT, we will investigate whether these differences are clinically and statistically significant among a large and diverse group of individuals, with respect to race, ethnicity, geography, and language, and determine whether these results are consistent across subgroups. We will also conduct exploratory analyses identifying patient-level characteristics, clinical factors (such as illness severity) or other social drivers of health associated with a greater treatment response. Identifying these factors will enable us to continue to fine-tune our approach in preparation for scaling the intervention to a population level, making it a ubiquitous resource for patients and their families during their transition after ICU discharge.

## Supplementary Material

MMC1

## Figures and Tables

**Table 1:** Two-stage inclusion and exclusion criteria.

Stage 1. Eligibility Review Prior to Approaching Patient for Potential Study Inclusion			

Condition	Hospital-based Inclusion Criteria	Condition	Hospital-based Exclusion Criteria

Age	Age ≥ 18	Drug/Alcohol Abuse	Active alcohol or drug abuse (e.g., admission for alcohol withdrawal, drug-related complication, positive toxicology screening at admission, endorsement of active addiction)
Medical Intervention (s)	Managed in an ICU or stepdown unit for ≥24 h during which they had a serious acute cardiorespiratory condition, defined as ≥1 of the following: ● mechanical ventilation via endotracheal tube for ≥4 h ● non-invasive ventilation (CPAP, BiPAP) for ≥4 h in a 24-h period provided for acute respiratory failure ● new use of supplemental oxygen ≥6 l per minute (or increase in baseline continuous oxygen) ● use of vasopressors or inotropes for shock of any etiology ● use of pulmonary vasodilators ● use of aortic balloon pump or cardiac assist device for cardiogenic shock ● use of diuretic intravenous drip ● evidence of acute coronary ischemia ● urgent cardiac catheterization	Complex Medical Needs after Discharge	Anticipated complex medical needs after discharge that would be disruptive to intervention and follow up, such as anticipated surgical procedures, complex medical regiments (e.g., new chemotherapy, new dialysis, pregnant and near term), or plans for comfort care
		Other complex needs post-discharge	Other complex needs anticipated that could interfere with the ability to complete study procedures, such as upcoming travel, inability to use the mobile app, and anticipated unstable living situations
		Living arrangements post-discharge	Anticipated or actual discharge to a location other than an independent home setting (e.g., nursing home, long-term acute care facility, inpatient rehabilitation facility, home hospice)
		Incarceration Technology constraints	Currently imprisoned or incarcerated or in home detention Lack of a reliable smartphone with cellular data plan or access to the internet
		Concurrent study participation	Concurrently enrolled in another study involving an intervention whose objectives conflict with the objectives of this study
		Prior enrollment	Previously enrolled in this study
Cognitive health	Intact cognitive status		Persistent impaired cognition as a result of illness, with impairment defined as ≥3 errors on the Callahan cognitive status screen and/or the lack of decisional capacity
Mental health	No treatment for severe mental illness within 6 months preceding the current hospital admission, no evidence of poorly managed severe mental illness, and no endorsement of suicidality at time of admission or informed consent		
Language	Functional fluency in English or Spanish		
Stage 2. Eligibility Review After Obtaining Consent			
	Post-discharge Inclusion Criteria		Post-discharge Exclusion Criteria
HADS score	HADS score ≥ 8	Failure to randomize	Failure to randomize within 14 days after discharge (due to not completing the HADS or receiving a score < 8)
		Hospital readmission	Readmitted to hospital (for >24 h) before randomization

**Table 2:** Outcomes and other measures collected by timepoint.

Outcome/assessment^a^	In hospital	Baseline	Intervention^b^	Follow up (months)		
				
				1	3	6
***Primary Outcome***						
Hospital Anxiety and Depression Scale (HADS)		X	X	X	X	X
***Secondary Outcomes***						
HADS anxiety subscale		X	X	X	X	X
HADS depression subscale		X	X	X	X	X
Posttraumatic Stress Scale (PTSS)		X		X	X	X
EQ-5D 100-point VAS quality of life		X		X	X	X
Modified Patient Health Questionnaire 15 physical symptom scale (mPHQ-15)		X		X	X	X
***Other Measures***						
Sociodemographics: age, gender, race, ethnicity, employment, insurance, education, marital status, children, social support	X					
Clinical characteristics: Medical comorbidities, illness severity, delirium during ICU stay, ICU diagnosis, ICU service, duration of ventilation, length of stay	X					
Perceived Stress Scale (PSS-4)		X		X	X	X
Perceived social support, financial stress, optimism, and hope		X		X	X	X
ADL and IADL Limitations		X		X	X	X
Psychiatric and other Medical Services Use		X		X	X	X
Intervention adherence app analytics (# of sessions, total time, most utilized elements)			X			

a See Supplementary Material Appendix B for detailed descriptions of outcomes and other study assessments.

b Assessed weekly during 4-week intervention.

## Data Availability

No data was used for the research described in the article.

## Appendix A. Supplementary data

Supplementary data to this article can be found online at https://doi.org/10.1016/j.cct.2026.108359.

## Glossary

AE: adverse event
APACHE II: Acute Physiology And Chronic Health Evaluation II
ARDS: acute respiratory distress syndrome
cLDA: constrained longitudinal data models
CRC: clinical research coordinator
CST: adaptive coping skills training
DCC: data coordinating center
DSMB: data safety monitoring board
EMR: electronic medical record
HADS: hospital anxiety and depression scale
ICU: intensive care unit
mPHQ-15: modified Patient Health Questionnaire-15
PICS: post-intensive care syndrome
PTSD: posttraumatic stress disorder
PTSS: posttraumatic stress scale
RCT: randomized clinical trial
SAE: serious adverse event
VAS: visual analog scale
