# Supplementary material for: Blueprint 2: A self-directed mobile adaptive coping skills intervention to improve psychological distress symptoms among cardiorespiratory failure survivors study protocol
Source: Contemp Clin Trials. Author manuscript; Available in PMC 2026 Aug 14. (PMC13475743; doi:10.1016/j.cct.2026.108359)
Supplement: Supplementary file 1 [file NIHMS2187520-supplement-MMC1.docx]

Supplemental Material

[Appendix A: Experimental condition allocation using method of minimization	2](#_Toc208307883)

[Appendix B: Detailed descriptions of study outcomes and other measures	2](#_Toc208307884)

[References	5](#_Toc208307885)

## Appendix A: Experimental condition allocation using method of minimization

Due to randomization occurring within app and separate apps used by English and Spanish speaking users, participants (n=400) will first be stratified by language. Within each language group, participants will be assigned with equal allocation into one of two conditions (Blueprint intervention or Education control) while minimizing imbalance across study site (Duke, UCLA, OHSU, Colorado) and anxiety and depression symptoms at baseline (T0 HADS total score <20 vs. ≥20) using the steps described below:

1. As each participant is ready to be randomized, calculate the marginal balance (*b_M_*) assuming a specific experimental condition assignment (i.e., condition 1) as detailed in section 2.4 of Han et al (2009)^1^ where *N=2* and *a_i_*=the number of participants for a factor level already assigned to each condition*, i=1,2*.
2. This calculation is repeated for each factor level, and the balance scores across factors are summed to create an overall score for that experimental condition assignment.
3. The procedure is repeated for each condition (once participants have been assigned to both groups) with the result of 2 overall marginal balance scores.
4. The participant is assigned to the experimental condition which results in the smallest marginal balance score (i.e., imbalance is minimized). If there is a tie between experimental conditions with the smallest marginal balance score, then the participant is assigned randomly to one of those tied experimental conditions.
5. The process is repeated for each consecutive participant enrolled.

## Appendix B: Detailed descriptions of study outcomes and other measures

Primary and secondary outcomes are described in further detail below:

- Hospital Anxiety and Depression Scale (HADS) is a self-assessment questionnaire with seven questions ranging 0-3 points each used to calculate anxiety and depression subscales, all 14 of which can be combined to calculate total score. Anxiety and Depression subscales are measures on a 0 to 21 scale, for which 0=lower limit of normal and 21=the upper limit of severe depression or anxiety and ranges within the subscale scores are clinically interpreted as follows: 0-7 normal (i.e., non-cases), 8-10 mild, and 11-14 moderate, and 15-21 severe.^37^ HADS total score is measures on a 0 to 42 scale.
- Post-Traumatic Stress Scale (PTSS) is a 10-item post-traumatic stress disorder (PTSD) symptom scale used frequently to assess ICU-related traumas by anchoring memory recall to hospitalization.^38^ Scoring for each question range from 1 for never to 7 for always and ask participants how often they have experienced the following in the past week: sleep problems, nightmares, depression or felt dejected/downtrodden, jumpiness, the need to withdraw from others, irritability, mood swings, bad conscience, fear of places or situations that remind them of the ICU, and muscular tension. The total score is calculated by adding questions scores and ranges from 10-70 with 10 and 70 indicated lowest and highest levels of PTSD measures, with scores ≥20 considered to be notably elevated.
- EQ-5D 100-point visual analog scale (VAS) quality of life scale is a VAS designed for self-completion that collects quality of life information on a 0-100 scale for which 0=worst imaginable health state and 100=best imaginable health state.
- Modified Patient Health Questionnaire-15 physical symptom scale (mPHQ-15) is a ten-item version of the PHQ-15 measure of physical symptoms^35^ that has been adapted for post-ICU patients and used previously by investigators in similar studies.^39^ The mPHQ-15 asks participants “Over the past week or so, how much have you been bothered by any of the following problems?” for the following symptoms: 1) Pain, 2) Difficulty concentrating, 3) Trouble falling asleep, 4) Weakness, 5) Feeling your heart pound or race, 6) Shortness of breath (or other breathing problems), 7) Stiffness in joints or muscles, 8) Feeling tired or having little energy, 9) Constipation, loose stools or diarrhea, 10) Nausea, gas, or indigestion. Scores for each question range from 0 (not bothered at all) to 2 (bothered a lot) and total scores calculated by adding question scores can range from 0 (best) to 20 (worst). Notes on this adapted version: PHQ-15 items n (feeling tired or having low energy) and o (trouble falling / staying asleep) are not included. Question 1 (Pain) is formed by collapsing 7 original PHQ-15 pain-themed items: a, b, c, d, e, f, and k (a: *stomach pain, b: back pain, c: pain in arms/legs/joints, d: menstrual cramps and other problems with your periods; women only, e: headaches, f: chest pain, g: pain or problems during sexual intercourse*). Question 2 (Difficulty concentrating) replaces 2 original PHQ-15 items (*g: dizziness & h: fainting spells*) because they don’t fit the population well. Question 7 (Stiffness in joints or muscles) is reworded from the original PHQ-15 item (c: pain in your arms, legs, or joints) to ‘stiffness’ for conciseness. Question 3 (Weakness) was also added.

Other study assessments are described in further detail below:

- The Perceived Stress Scale (PSS-4)^40^ asks participants four questions related to stress in the last month:
  - In the last month, how often have you felt that you were unable to control the important things in your life? 2. In the last month, how often have you felt confident about your ability to handle your personal problems? 3. In the last month, how often have you felt that things were going your way? 4. In the last month, how often have you felt difficulties were piling up so high that you could not overcome them? Scoring Instructions: Total score is determined by adding together the scores of each of the four items. Questions 2 and 3 are reverse coded. Questions 1 and 4: 0 = Never; 1 = Almost never; 2 = Sometimes; 3 = Fairly often; 4 = Very often Questions 2 and 3: 4 = Never; 3 = Almost never; 2 = Sometimes; 1 = Fairly often; 0 = Very often
- Questions developed for this study and answer ranges or options [with score] listed below:
  - I have someone to confide in, or talk to about myself or my problems” Never [5] – Always [1]
  - “At the end of an average month, what describes how you usually feel when you were paying the bills?” Short on money and need more to pay bills [4] Barely have enough to pay bills and for basic needs [3] Have enough for just a few extra things [2] Completely comfortable [1]
  - Overall, I expect more good things to happen to me than bad. Strongly Agree [5] – Strongly Disagree [1]
  - In the past week or so, I felt hopeful. Never [5] – Always [1]
- The following ADL and IADL Limitations questions were adapted for this study from those published by Katz et al^41^ and Lawton et al^42^ with answer options to the questions below ranging from “Need no help” (0) to “Need some help or unable to do” (1) with a composite score calculated as the sum of these answers. The questions are as follows: “Currently I am able to: 1) … bathe myself (either a sponge bath, tub bath, or shower) , 2) …dress myself (like putting on a shirt, buttoning and zipping, etc), 3) …feed myself (holding fork, cutting food, drinking from a glass), 4) …get from a bed to a chair by myself, 5) …control my bladder or bowels, 6) …use toilet by myself, 7) …use the telephone (including looking up numbers and dialling), 8) …get to places beyond walking distance (drive own car or travel alone in buses or taxis), 9) …go shopping for groceries or clothes, 10) …prepare my own meals, 11) …do housework (wash dishes, clean floors, etc.), 12) …take my own medicine, 13) …handle my own money (write checks, pay bills)
- Additional Psychiatric and other Medical Services Use variables collected through the Pattern Health at all timepoints include: currently being treated by medication or mental health specialist for Depression, Anxiety, PTSD, or Chronic pain and whether they were readmitted to the hospital after discharge and if so, how many times.

## References

1.	Han B, Enas NH and McEntegart D. Randomization by minimization for unbalanced treatment allocation. *Stat Med* 2009; 28: 3329-3346. 2009/09/10. DOI: 10.1002/sim.3710.
